# Probabilistic Communication-State Inference for Agricultural Robots Under Wireless Degradation

**DOI:** 10.3390/s26123937

**Published:** 2026-06-21

**Authors:** Donghee Noh, Hea-Min Lee

**Affiliations:** AI Application Research Center, Jeonbuk Regional Branch, Korea Electronics Technology Institute (KETI), Jeon-Ju 54853, Republic of Korea; lee10849@keti.re.kr

**Keywords:** agricultural robots, remote supervision, communication-state inference, wireless link reliability, probabilistic degradation modeling, contextual uncertainty, smart greenhouse, RSSI, PRR

## Abstract

Remote supervision of agricultural robots depends on continuous interpretation of robot status and wireless link quality. In smart greenhouses, crop canopies, metallic frames, cultivation rows, and non-line-of-sight propagation can cause intermittent packet loss and RSSI attenuation. Treating such transient degradation as immediate communication failure can interrupt robot operation unnecessarily, whereas delayed recognition of persistent loss can compromise safety. This study proposes a probabilistic communication-state inference method for remotely supervised agricultural robots. The robot-to-gateway wireless link is represented by three states: normal, degraded, and failure. The degraded state acts as an uncertainty buffer that preserves recoverable degradation before failure escalation. Packet reception ratio, received signal strength, and trajectory-derived context are used to update state probabilities through a bounded transition mechanism. Field experiments with a mobile agricultural robot in a smart greenhouse showed an accuracy of 0.915±0.007 and a macro F1-score of 0.907±0.008, while reducing the premature failure rate to 18.0±1.4%. Comparisons with threshold-based, moving-average, and adapted WSN fault-detection baselines, including a FedLSTM-inspired baseline, showed that binary fault-detection logic cannot explicitly preserve recoverable degraded communication intervals. The results indicate that probabilistic degradation modeling supports communication-aware remote supervision by distinguishing transient degradation from failure-level communication loss.

## 1. Introduction

Agricultural robots are increasingly deployed in smart greenhouses, orchards, and open-field workspaces for navigation, crop monitoring, spraying, harvesting, and transport [1,2]. These robots are becoming part of cyber-physical farming systems in which sensing, communication, data processing, irrigation management, and human supervision are tightly coupled [3,4,5,6,7,8]. In remotely supervised operation, wireless communication carries robot status, control messages, safety notifications, and monitoring data. Link reliability is therefore an operational requirement, not only a network-performance metric.

**Figure 1:**
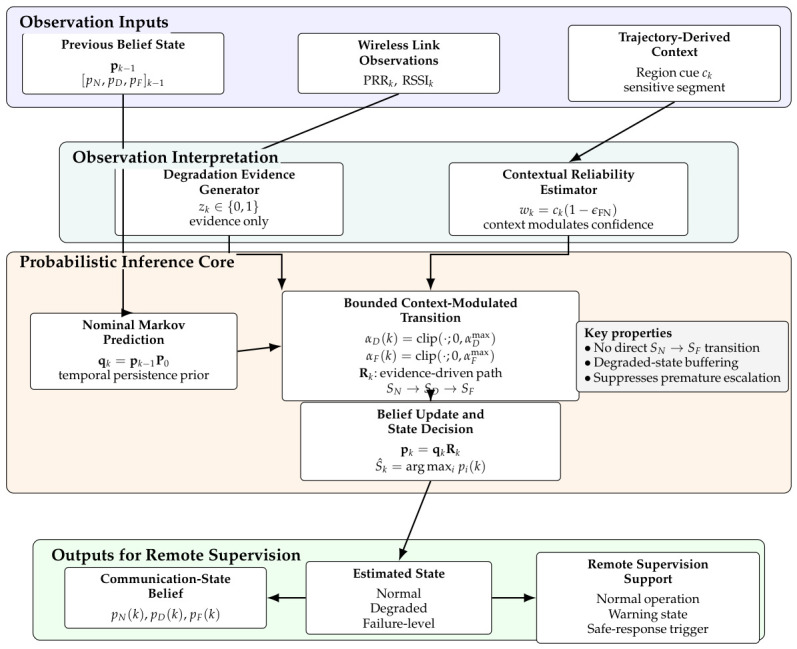
Architecture of the proposed probabilistic communication-state inference framework. Wireless link observations, ${\mathrm{PRR}}_{k}$ and ${\mathrm{RSSI}}_{k}$, are converted into degradation evidence $z_{k}$, while trajectory-derived context is mapped to a contextual reliability weight $w_{k}$. The previous belief state is propagated through the nominal Markov prior $P_{0}$ and then updated by the bounded context-modulated transition operator $R_{k}$, which introduces degraded-state buffering and suppresses premature escalation to the failure state.

Figure 1 summarizes the proposed probabilistic communication-state inference framework. The framework separates wireless-link observation interpretation from probabilistic state updating: PRR and RSSI are first converted into degradation evidence, trajectory-derived context is mapped to a contextual reliability weight, and the previous belief state is then updated through nominal Markov prediction and a bounded context-modulated transition operator.

Wireless links in agricultural environments are unstable because crop canopies, metallic frames, cultivation beds, robot motion, gateway placement, and non-line-of-sight propagation affect signal quality [5,8]. Recent teleoperation and field-network studies show that agricultural wireless networks can support remote robot operation, but they also report reliability, latency, and field-dependent variability issues [9,10]. In smart greenhouses, dense vegetation, narrow aisles, irrigation facilities, and metal structures can produce packet loss and RSSI attenuation even when the robot and communication hardware are functioning normally.

Remote supervision requires a distinction between transient degradation and persistent failure. Immediate failure decisions under short-term packet loss can stop the robot unnecessarily and increase operator workload. Conversely, delayed recognition of persistent communication loss can allow the robot to operate without reliable supervision. A practical monitoring method must therefore represent normal communication, recoverable degradation, and failure-level communication loss as different operational states.

Conventional monitoring commonly uses packet reception ratio (PRR), received signal strength indicator (RSSI), packet loss, or cyclic redundancy check outcomes [5,11]. These measurements are simple and suitable for embedded platforms, but direct thresholding can be unstable for mobile robots. A PRR or RSSI decrease may indicate failure, but it may also reflect temporary propagation effects caused by crop occlusion, robot position, gateway geometry, or local structures. Moving averages reduce abrupt state changes but can delay detection and still provide no explicit representation of uncertainty between normal and failure states.

Learning-based and model-based approaches have been proposed for wireless sensor network (WSN) fault diagnosis, including ensemble learning, Kalman filtering, belief-rule-base reasoning, and federated LSTM models [12,13,14,15]. These methods are effective for sensor-node or data-fault detection when suitable training data and computing resources are available. However, communication-state inference for robot supervision is different: the system must determine whether the robot remains sufficiently connected for supervision, whether the link is degraded but recoverable, or whether the communication condition requires safety-oriented response.

This study proposes a probabilistic degradation-aware communication-state inference method for remotely supervised agricultural robots. The method represents the wireless link using three states: normal, degraded, and failure. The degraded state is introduced as an intermediate uncertainty buffer that prevents temporary packet loss or RSSI attenuation from being treated as immediate failure. PRR and RSSI provide communication evidence, while trajectory-derived context modulates transition confidence without acting as a deterministic label.

The main contributions are summarized as follows:

- Wireless communication monitoring for agricultural robots is reformulated as a three-state probabilistic inference problem, in which the degraded state serves as an explicit operational buffer between normal communication and failure-level communication loss.
- A bounded context-modulated transition mechanism is developed to combine PRR, RSSI, and trajectory-derived context while preventing contextual information or instantaneous link degradation from directly forcing a failure decision.
- The proposed method is designed as a lightweight inference framework that uses fixed-size belief updates without model training, making it suitable for embedded remote-supervision systems.
- The framework is validated through controlled simulations and repeated greenhouse field runs, and is compared with threshold-based, moving-average, and adapted WSN fault-detection baselines, including a FedLSTM-inspired baseline.

## 2. Background and Related Work

### 2.1. Communication Reliability in Remotely Supervised Agricultural Robots

Wireless communication is a core component of remotely supervised agricultural robot systems. In smart greenhouses, orchards, and other structured agricultural environments, mobile robots operate within cyber-physical farming systems that connect sensing, navigation, control, data processing, and human supervision through wireless networks [5,6,7]. Recent studies on agricultural robot teleoperation and field-network evaluation indicate that communication infrastructure directly affects remote monitoring, operator intervention, and safe robot operation [9,10].

Conventional agricultural IoT and WSN systems commonly assess link reliability using PRR, RSSI, packet loss, and error-check outcomes [5,11,13]. These indicators are simple and practical, but their interpretation changes when the node is a mobile robot. In static networks, repeated packet errors may indicate a link or node fault. In a moving greenhouse robot, the same observations may result from crop occlusion, metallic structures, gateway geometry, or local non-line-of-sight propagation. Treating all such events as persistent failures can cause unnecessary robot stops, whereas ignoring persistent loss can compromise supervision.

Agricultural environments further complicate link interpretation. In greenhouses, crop density, metallic frames, cultivation beds, irrigation facilities, robot trajectory, and local propagation conditions affect wireless quality. In orchards and open fields, terrain, crop rows, vegetation, and gateway placement can create similar variability [8,9,10]. Communication monitoring for agricultural robots should therefore infer operational communication states rather than only threshold instantaneous link-quality measurements.

### 2.2. Data-Driven Communication-State Estimation: Strengths and Limitations

Machine learning, statistical filtering, ensemble learning, and federated learning have been applied to WSN fault detection and diagnosis. Extra-Trees-based classifiers have been used to classify multiple WSN fault types [12]. Kalman-filter-based approaches have been investigated for sensor-fault detection in smart irrigation networks [13]. More recent belief-rule-base and federated LSTM frameworks address uncertain or distributed WSN fault-diagnosis settings [14,15]. These methods can capture complex fault patterns when sufficient labeled data and computing resources are available.

Their objective, however, differs from that of agricultural robot communication-state inference. Most WSN fault-diagnosis methods identify faulty nodes, abnormal readings, or hardware-related data faults after abnormal patterns appear. Remote robot supervision requires an operational interpretation of the link: whether the robot remains connected for supervision, whether the link is degraded but recoverable, or whether the condition requires operator intervention or safe-mode transition.

Directly transferring learning-based fault diagnosis to agricultural robot communication monitoring has practical limits. Embedded robot platforms must also run perception, navigation, and control, which constrains computation and memory. In addition, labeled communication datasets covering crop growth stages, greenhouse layouts, robot paths, gateway placements, and local obstacles are difficult to collect. Many data-driven methods also classify abnormality rather than model state transitions; transient environmental degradation may therefore be treated as a fault even when the link has not failed. A lightweight and interpretable method is needed to represent uncertainty during recoverable degradation while remaining sensitive to persistent communication loss.

### 2.3. Degradation-Aware Probabilistic State Modeling

Probabilistic state models are suitable for communication-state inference because they represent state persistence, transition dynamics, and uncertainty with low computational cost. Markov, Bayesian, and hybrid reasoning frameworks have been used in WSN and cyber-physical monitoring to improve robustness under noisy observations [14,16,17,18]. These properties are relevant because PRR and RSSI fluctuate under environmental propagation effects and do not always indicate persistent failure.

Existing probabilistic and learning-based fault-diagnosis methods generally focus on sensor-node faults or abnormal data patterns. They do not explicitly model the intermediate operational state in which a mobile robot experiences degraded but recoverable communication during remote supervision. Context-aware frameworks incorporate auxiliary information such as topology, link-quality estimates, or cross-layer features [19,20], but contextual signals are often treated as direct inputs or deterministic indicators. In agricultural environments, context may be incomplete, delayed, coarse, or only indirectly related to link quality. It should therefore modulate transition confidence rather than directly determine the communication state.

To address these constraints, the proposed framework treats PRR and RSSI as communication evidence and trajectory-derived context as an uncertainty-modulating signal. The wireless link is modeled using normal, degraded, and failure states. The degraded state accumulates uncertain evidence before failure escalation, reducing premature failure decisions while preserving responsiveness to persistent communication loss.

## 3. System Architecture and Problem Formulation

### 3.1. System Architecture Overview

The target system is a remotely supervised agricultural robot operating in a structured agricultural environment. The robot exchanges status, monitoring data, safety notifications, and control-related information with a supervision system through a wireless robot-to-gateway link. Human operators can intervene when abnormal or unsafe conditions occur; therefore, link reliability directly affects operational continuity and safety.

The robot moves through crop rows, greenhouse structures, narrow aisles, and communication-sensitive regions. Crop canopies, metallic frames, cultivation beds, irrigation facilities, and robot motion may cause temporary packet loss, RSSI attenuation, or non-line-of-sight-like degradation even when the communication hardware is normal. The system therefore requires an inference mechanism that distinguishes normal communication, recoverable degradation, and failure-level communication loss.

The proposed framework combines link-quality observations with trajectory-derived context. PRR and RSSI provide primary communication evidence. Context indicates whether the robot is in a region where degradation is likely, but it is not treated as a deterministic failure label. Instead, context modulates the confidence of probabilistic state transitions under uncertainty. The objective is to estimate the latent communication state over time for remote supervision, not to diagnose permanent sensor-node faults.

### 3.2. Communication-State Inference Problem

At each discrete time step *k*, the latent communication state of the robot-to-gateway wireless link is defined as

(1) $$S_{k}\in \left\{S_{N},S_{D},S_{F}\right\},$$

where $S_{N}$, $S_{D}$, and $S_{F}$ denote the normal, degraded, and failure-level communication states, respectively. For readability, $S_{F}$ is hereafter referred to as the failure state unless further distinction is required.

The normal state represents a condition in which the wireless link quality is sufficient for remote supervision. The degraded state represents a recoverable but uncertain condition in which link quality is temporarily reduced but not sufficient to justify failure-level classification. The failure state represents persistent communication loss or severe link degradation that may require a robot stop, safe-mode transition, or operator intervention.

The degraded state plays a central role as an operational uncertainty buffer between normal communication and failure-level disconnection. It captures situations in which communication behavior is abnormal but the evidence is insufficient to determine that the link has failed. This design prevents premature escalation to the failure state under transient disturbances caused by robot motion, local NLoS-like propagation, gateway shadowing, or temporary crop occlusion.

The belief over the communication states at time step *k* is represented as

(2) $$p_{k}=\left[p_{N}\left(k\right),\;p_{D}\left(k\right),\;p_{F}\left(k\right)\right],$$

where $p_{N}\left(k\right)$, $p_{D}\left(k\right)$, and $p_{F}\left(k\right)$ denote the probabilities of the normal, degraded, and failure states, respectively. The belief vector satisfies

(3) $$p_{i}\left(k\right)\geq 0,\;\sum_{i\in \{N,D,F\}}p_{i}\left(k\right)=1.$$

The key mathematical symbols and variables used in the proposed communication-state inference framework are summarized in Table 1.

The discrete communication-state estimate used for evaluation is obtained by the maximum-belief rule,

(4) $${\hat{S}}_{k}=\arg\max_{S_{i}\in \left\{S_{N},S_{D},S_{F}\right\}}p_{i}\left(k\right).$$

The full belief vector is retained during inference because it provides uncertainty information for remote supervision and safety decision-making. Thus, the proposed method does not directly convert a single communication measurement into a failure decision. Instead, it sequentially updates the communication-state belief so that temporary degradation can be buffered in the degraded state before the model escalates to the failure state.

### 3.3. Degradation Evidence from Link-Quality Observations

The communication degradation indicator $z_{k}\in \left\{0,1\right\}$ is constructed from PRR and RSSI measurements. It represents instantaneous evidence of link degradation, not the final communication-state label. In this study, $z_{k}$ is defined as

(5) $$z_{k}=\left\{\begin{array}{cc} 1, & \mathrm{if}\;{\mathrm{PRR}}_{k}<\theta_{\mathrm{PRR}}\;\mathrm{or}\;{\mathrm{RSSI}}_{k}<\theta_{\mathrm{RSSI}},\\ 0, & \mathrm{otherwise}, \end{array}\right.$$

where $\theta_{\mathrm{PRR}}$ and $\theta_{\mathrm{RSSI}}$ are empirical thresholds used only to construct degradation evidence. These thresholds are not used to directly assign the final state among $S_{N}$, $S_{D}$, and $S_{F}$. The final state estimate is determined by the subsequent probabilistic belief update described in Section 3.5 and Section 3.6, rather than by the threshold rule itself.

When $z_{k}=1$, the current communication observation provides evidence of reduced link quality, such as packet loss, RSSI attenuation, or abnormal packet-level behavior. When $z_{k}=0$, the observation does not provide strong evidence of communication degradation. This formulation separates low-level link-quality evidence from high-level operational communication-state inference.

### 3.4. Trajectory-Derived Contextual Reliability

The contextual indicator $c_{k}\in \left\{0,1\right\}$ represents whether the robot is located in a trajectory region where communication degradation is more likely to occur. Examples include crop-dense regions, metallic structure-dense regions, narrow greenhouse corridors, or path segments where NLoS-like propagation is likely. However, such contextual information is imperfect and should not be interpreted as a direct communication-state label. A communication-sensitive region may not always cause degradation, and degradation may also occur outside such a region.

To represent contextual observation errors, the following probabilities are defined:

(6) $$\mathrm{Pr}\left(c_{k}=0|C_{k}=1\right)=\epsilon_{\mathrm{FN}},\;\mathrm{Pr}\left(c_{k}=1|C_{k}=0\right)=\epsilon_{\mathrm{FP}},$$

where $C_{k}$ denotes the latent condition that the robot is located in a communication-sensitive context. Here, $\epsilon_{\mathrm{FN}}$ represents the false-negative rate of contextual observation, and $\epsilon_{\mathrm{FP}}$ represents the false-positive rate. This formulation acknowledges that contextual observations are uncertain and should not be used to directly determine the communication state.

Although both $\epsilon_{\mathrm{FN}}$ and $\epsilon_{\mathrm{FP}}$ characterize contextual observation errors, the proposed transition modulation primarily depends on missed communication-sensitive contexts represented by $\epsilon_{\mathrm{FN}}$. This is because false-negative contextual observations directly reduce the contextual reliability weight and weaken the buffering effect of the degraded state. Therefore, $\epsilon_{\mathrm{FN}}$ is treated as the main contextual uncertainty parameter in the sensitivity analysis, while $\epsilon_{\mathrm{FP}}$ is retained to describe the general uncertainty model of contextual observation.

The contextual reliability weight is defined as

(7) $$w_{k}=c_{k}\left(1-\epsilon_{\mathrm{FN}}\right),$$

where $w_{k}\in \left[0,1\right]$. A larger $w_{k}$ indicates that the observed communication degradation is more likely to be associated with a known communication-sensitive trajectory region. A smaller $w_{k}$ indicates insufficient contextual support for interpreting the degradation as temporary or trajectory-induced. In the proposed framework, $w_{k}$ modulates the transition process rather than directly classifying the communication state.

### 3.5. Nominal Markov Prediction

Before incorporating degradation evidence and contextual reliability, the state belief evolves according to a nominal Markov transition prior,

(8) $$q_{k}=p_{k-1}P_{0},$$

where $P_{0}\in R^{3\times 3}$ is a row-stochastic baseline transition matrix and $q_{k}$ denotes the predicted belief before evidence-driven modulation. The matrix $P_{0}$ encodes the temporal persistence of communication states between consecutive communication observations.

Because the field communication data in this study were sampled at 1 Hz, the diagonal terms of $P_{0}$ were set close to one. This design prevents evidence-free state drift, in which the belief would move toward the degraded or failure state even when no degradation evidence is observed. The off-diagonal terms allow gradual state evolution while preserving temporal persistence over consecutive observations.

The nominal transition matrix used in this study is given by

(9) $$P_{0}=\left[\begin{array}{ccc} 0.995 & 0.005 & 0.000\\ 0.005 & 0.990 & 0.005\\ 0.000 & 0.005 & 0.995 \end{array}\right].$$

The direct transition probability from the normal state to the failure state is set to zero in the nominal prior because failure-level link loss is assumed to be preceded by a degraded communication interval within the sampling horizon considered in this study. Similarly, the direct transition from the failure state to the normal state is not allowed in a single step because recovery from failure-level communication loss should require sufficient recovery evidence or supervisory confirmation.

The nominal transition matrix is not intended to represent a universal communication-state transition law. Rather, it serves as a temporal persistence prior for the 1 Hz communication-state update. To avoid treating this prior as an arbitrary fixed setting, the influence of alternative transition matrices is examined through sensitivity analysis in Section 5.7.

### 3.6. Bounded Context-Modulated Transition Operator

After the nominal Markov prediction, communication degradation evidence and contextual reliability are incorporated through a bounded transition operator. The proposed update uses two bounded transition coefficients: one for normal-to-degraded probability transfer and one for degraded-to-failure probability transfer.

The normal-to-degraded transition coefficient is defined as

(10) $$\alpha_{D}\left(k\right)=\mathrm{clip}\left(\gamma_{D}z_{k}\left(1+\lambda w_{k}\right),0,\;\alpha_{D}^{\max}\right),$$

where $\gamma_{D}$ is the degradation-transition coefficient, λ is the context-modulation scaling factor, and $\alpha_{D}^{\max}$ is the upper bound on the normal-to-degraded transition coefficient. The operator clip(x,a,b) limits *x* to the interval [a,b].

The degraded-to-failure transition coefficient is defined as

(11) $$\alpha_{F}\left(k\right)=\mathrm{clip}\left(\gamma_{F}z_{k}\left(1-w_{k}\right),0,\;\alpha_{F}^{\max}\right),$$

where $\gamma_{F}$ is the failure-escalation coefficient and $\alpha_{F}^{\max}$ is the upper bound on the degraded-to-failure transition coefficient.

The bounded context-modulated transition operator is then defined as

(12) $$R_{k}=\left[\begin{array}{ccc} 1-\alpha_{D}\left(k\right) & \alpha_{D}\left(k\right) & 0\\ 0 & 1-\alpha_{F}\left(k\right) & \alpha_{F}\left(k\right)\\ 0 & 0 & 1 \end{array}\right].$$

The updated belief is computed as

(13) $$p_{k}=q_{k}R_{k}=p_{k-1}P_{0}R_{k}.$$

This formulation makes the role of the degraded state explicit. Since the $\left(S_{N},S_{F}\right)$ element of $R_{k}$ is zero, degradation evidence cannot directly transfer probability mass from the normal state to the failure state. Instead, the evidence first increases the degraded-state probability. Failure probability increases only through the degraded state and only according to the bounded escalation coefficient $\alpha_{F}\left(k\right)$.

### 3.7. Interpretation of Contextual Modulation

The contextual reliability weight $w_{k}$ has opposite effects on degraded-state buffering and failure escalation. From Equations (10) and (11), when the clipping bounds are inactive, the following monotonic relationships hold:

(14) $$\frac{\partial \alpha_{D}\left(k\right)}{\partial w_{k}}=\gamma_{D}z_{k}\lambda \geq 0,$$

and

(15) $$\frac{\partial \alpha_{F}\left(k\right)}{\partial w_{k}}=-\gamma_{F}z_{k}\leq 0.$$

Therefore, increasing contextual reliability strengthens the transfer of probability mass from the normal state to the degraded state, while suppressing escalation from the degraded state to the failure state. This behavior formalizes the intended operational interpretation: if communication degradation occurs in a trajectory region where temporary attenuation is expected, the model buffers the evidence in the degraded state rather than immediately increasing the failure probability.

When $c_{k}=1$, the contextual reliability weight becomes $w_{k}=1-\epsilon_{\mathrm{FN}}$. In this case, the degraded-to-failure coefficient becomes

(16) $$\alpha_{F}\left(k\right)=\mathrm{clip}\left(\gamma_{F}z_{k}\epsilon_{\mathrm{FN}},0,\;\alpha_{F}^{\max}\right).$$

Thus, if the contextual observation is reliable and $\epsilon_{\mathrm{FN}}$ is small, premature escalation to the failure state is strongly suppressed. Conversely, when $c_{k}=0$ or when contextual support is unreliable, $w_{k}$ becomes small and persistent degradation evidence can gradually increase the failure-state probability.

### 3.8. Analytical Properties of the Proposed Update

The proposed belief update has three analytical properties that are useful for communication-state inference under uncertain wireless degradation.

First, the update preserves a valid probability distribution. If $p_{k-1}$ is a valid probability vector, $P_{0}$ is row-stochastic, and $0\leq \alpha_{D}\left(k\right),\alpha_{F}\left(k\right)\leq 1$, then $R_{k}$ is also row-stochastic. Therefore, $p_{k}=p_{k-1}P_{0}R_{k}$ remains non-negative and sums to one. This avoids ad hoc probability correction and ensures that the belief vector remains probabilistically interpretable.

Second, the evidence-driven update prevents direct normal-to-failure escalation. Because $R_{NF}\left(k\right)=0$, communication degradation evidence must first pass through the degraded state before increasing the failure-state probability. This structure implements the degraded state as an explicit uncertainty buffer between normal communication and failure-level communication loss.

Third, failure escalation is bounded. From Equation (11), the degraded-to-failure transition coefficient satisfies

(17) $$0\leq \alpha_{F}\left(k\right)\leq \alpha_{F}^{\max}.$$

Consequently, the increase in failure-state probability at a single time step is limited by the bounded transition coefficient. This prevents abrupt failure decisions caused by transient PRR reduction or RSSI attenuation, while still allowing failure probability to increase when degradation persists without sufficient contextual support.

Together, these properties distinguish the proposed method from direct threshold-based classification and moving-average-based smoothing. The proposed method treats communication degradation as sequential probabilistic evidence, preserves uncertainty in the degraded state, and controls failure escalation through a bounded context-modulated transition operator.

## 4. Proposed Probabilistic Communication-State Inference Algorithm

This section presents the proposed probabilistic communication-state inference algorithm based on the degradation-aware state formulation introduced in Section 3. The algorithm integrates communication-level observations and trajectory-derived context to estimate the wireless link state of a remotely supervised agricultural robot under intermittent and uncertain propagation conditions.

### 4.1. Design Principles

The proposed algorithm is designed according to the following principles.

First, communication-state inference is formulated as a probabilistic belief update problem rather than as a binary failure decision process. Temporary communication anomalies are not immediately interpreted as failure-level disconnection. Instead, uncertainty is preserved through the degraded state and gradually resolved through sequential probability updates.

Second, communication degradation serves as the primary evidence for state inference. Packet-level and link-quality indicators, such as packet reception ratio (PRR), received signal strength indicator (RSSI), and abnormal packet reception behavior, are used to construct the communication degradation indicator $z_{k}$.

Third, trajectory-derived context is incorporated as an uncertainty-modulating signal rather than as direct evidence of failure. The contextual indicator reflects whether the robot is located in a communication-sensitive region, such as a crop-dense path segment, a structure-dense greenhouse area, or a local propagation-sensitive zone. However, this information is treated as noisy and indirect; it modulates the confidence of interpreting communication degradation but does not deterministically determine the communication state.

Fourth, the algorithm is designed for lightweight execution on embedded agricultural robot platforms. All computations are based on fixed-size probability vectors and bounded scalar updates. The method does not require model training, backpropagation, large memory storage, or high-performance computing hardware.

### 4.2. Adaptive Inference Mechanism

The adaptive behavior of the proposed algorithm emerges from the interaction between communication evidence and contextual uncertainty.

When no communication degradation is observed, i.e., $z_{k}=0$, the state belief evolves according to the baseline Markov transition model. This reflects nominal state persistence and gradual temporal evolution under normal operating conditions.

When communication degradation is observed, i.e., $z_{k}=1$, the algorithm does not immediately classify the condition as a failure state. Instead, the belief is first shifted toward the degraded state, which represents a recoverable but uncertain communication condition. This allows the model to absorb transient packet loss or RSSI attenuation without causing premature escalation to failure-level communication loss.

Trajectory-derived context then modulates how the observed degradation is interpreted. If the contextual weight $w_{k}$ is high, the degradation is more likely to be associated with temporary environmental or trajectory-related propagation effects. In this case, probability mass is preferentially buffered in the degraded state, and escalation to the failure state is suppressed. If $w_{k}$ is low and degradation persists, the model gradually increases the failure probability because the degradation is less likely to be explained by transient contextual conditions.

This mechanism enables the proposed algorithm to distinguish transient communication degradation from persistent failure-level communication loss. As a result, the algorithm can reduce unnecessary robot stops and excessive operator interventions while maintaining responsiveness to communication conditions that may compromise safe remote supervision.

### 4.3. Algorithm Description

Algorithm 1 summarizes the proposed bounded context-modulated communication-state inference procedure. At each time step, the algorithm first converts PRR and RSSI measurements into degradation evidence and computes the contextual reliability weight from the trajectory-derived contextual indicator. It then performs nominal Markov prediction using $P_{0}$ and applies the bounded transition operator $R_{k}$. Because $R_{k}$ does not allow direct evidence-driven transition from $S_{N}$ to $S_{F}$, degradation evidence is first buffered in the degraded state before failure escalation occurs. The final output is the updated belief vector $p_{k}$, while the discrete state estimate ${\hat{S}}_{k}$ is used only for evaluation or supervisory decision support.
**Algorithm 1** Bounded context-modulated communication-state inference algorithm. **Require:**
Initial belief $p_{0}$, nominal transition matrix $P_{0}$, coefficients $\gamma_{D}$ and $\gamma_{F}$, context scaling factor λ, uncertainty parameter $\epsilon_{\mathrm{FN}}$, transition bounds $\alpha_{D}^{\max}$ and $\alpha_{F}^{\max}$ **Ensure:**
Communication-state belief $p_{k}$ and estimated state ${\hat{S}}_{k}$  1:**for** each time step *k* **do**  2:    Acquire link-quality observations ${\mathrm{PRR}}_{k}$ and ${\mathrm{RSSI}}_{k}$.  3:    Acquire trajectory-derived contextual indicator $c_{k}$.  4:    Set $z_{k}\leftarrow 1$ if ${\mathrm{PRR}}_{k}<\theta_{\mathrm{PRR}}$ or ${\mathrm{RSSI}}_{k}<\theta_{\mathrm{RSSI}}$; otherwise set $z_{k}\leftarrow 0$.  5:    Compute contextual reliability weight:$$w_{k}\leftarrow c_{k}\left(1-\epsilon_{\mathrm{FN}}\right)\;.$$  6:    Perform nominal Markov prediction:$$q_{k}\leftarrow p_{k-1}P_{0}\;.$$  7:    Compute the bounded normal-to-degraded transition coefficient:$$\alpha_{D}\left(k\right)\leftarrow \mathrm{clip}\left(\gamma_{D}z_{k}\left(1+\lambda w_{k}\right),0,\alpha_{D}^{\max}\right)\;.$$  8:    Compute the bounded degraded-to-failure transition coefficient:$$\alpha_{F}\left(k\right)\leftarrow \mathrm{clip}\left(\gamma_{F}z_{k}\left(1-w_{k}\right),0,\alpha_{F}^{\max}\right)\;.$$  9:    Construct the bounded context-modulated transition operator:$$R_{k}\leftarrow \left[\begin{array}{ccc} 1-\alpha_{D}(k) & \alpha_{D}(k) & 0\\ 0 & 1-\alpha_{F}(k) & \alpha_{F}(k)\\ 0 & 0 & 1 \end{array}\right].$$ 10:    Update the communication-state belief:$$p_{k}\leftarrow q_{k}R_{k}\;.$$ 11:    Estimate the discrete communication state:$${\hat{S}}_{k}\leftarrow \arg\max_{S_{i}\in \left\{S_{N},S_{D},S_{F}\right\}}p_{i}\left(k\right).$$ 12:**end for**

The algorithm is applied sequentially to the robot communication link during operation. Because the state vector has a fixed dimension and the update rules consist of a fixed-size Markov prediction and a bounded transition-operator multiplication, the computational cost per update step is constant. Therefore, the method is suitable for real-time deployment on embedded agricultural robot platforms.

Algorithm 1 itself does not require simulation-specific temporal constraints such as $\tau_{\lim}$, $\tau_{\mathrm{rec}}$, or $e_{m}$. These parameters are used only to generate controlled degradation scenarios in the simulation study and are not required when applying the proposed inference algorithm to field data.

### 4.4. Computational Complexity

The proposed algorithm is designed for real-time execution on resource-constrained embedded agricultural robot platforms.

Each update step consists of a fixed-size Markov prediction, construction of the bounded transition operator, and multiplication of two three-dimensional probability vectors and matrices. Because the number of states is fixed, the computational complexity per update step is O(1). The memory requirement is also constant because the algorithm only stores the current belief vector, the nominal transition matrix, the bounded transition operator, and scalar parameters.

Unlike learning-based approaches, the proposed method does not require offline training, iterative optimization, backpropagation, or large model storage. This makes it suitable for continuous communication-state monitoring on edge devices integrated into agricultural robots, where perception, navigation, control, and communication monitoring must operate simultaneously.

### 4.5. Relation to Remote Supervision Objectives

The objective of the proposed algorithm is not merely to maximize instantaneous classification accuracy, but to support stable and safe communication-state interpretation for remotely supervised agricultural robots.

In practical remote supervision scenarios, premature failure decisions can unnecessarily interrupt robot operation, trigger excessive operator intervention, or reduce the practicality of agricultural robot services. Therefore, the proposed method prioritizes reducing premature escalation to failure-level communication loss under transient communication degradation. This is achieved by preserving uncertainty in the degraded state before increasing the failure probability.

At the same time, persistent communication degradation that cannot be sufficiently explained by context is gradually interpreted as failure-level communication loss. This allows the algorithm to remain responsive to conditions in which the robot may no longer be reliably supervised through the wireless link. Thus, the proposed framework balances robustness against transient degradation with sensitivity to persistent communication failure.

The proposed inference algorithm does not explicitly impose deterministic temporal constraints such as fixed dwell times or threshold-based delay rules. Instead, temporal robustness emerges from probabilistic state evolution and degraded-state buffering. For evaluation purposes, additional temporal constraints may be introduced in the simulation or experimental setting to emulate realistic robot motion, communication sampling intervals, and supervision policies.

## 5. Simulation Results

This section evaluates the proposed probabilistic communication-state inference method under controlled communication uncertainty. The simulation represents a remotely supervised agricultural robot that experiences RSSI attenuation, degradation persistence, and uncertain trajectory-derived context while moving through communication-sensitive regions.

The simulation is not intended to emulate physical sensor-node faults. Instead, it tests whether degraded-state buffering can distinguish transient link degradation from failure-level communication loss. Kinematic-aware quantities such as the dynamic time threshold $\tau_{\lim}\left(v\right)$, recovery threshold $\tau_{\mathrm{rec}}$, and maximum error count $e_{m}$ are used only to generate controlled degradation and recovery patterns in the simulation; they are not part of the core inference model in Section 3 and Section 4. These simulation constraints emulate the fact that wireless degradation in agricultural robot operation often persists for several consecutive samples while the robot passes through crop-dense, rack-dense, or structure-dense segments, and then recovers after the robot exits the obstructed region. Thus, they provide controlled persistence and recovery patterns for evaluating whether the proposed inference model can distinguish recoverable degradation from failure-level communication loss under trajectory-dependent propagation changes.

### 5.1. Simulation Setup

The simulation settings and default inference parameters are presented before the result figures so that each metric can be traced to the assumptions used in the analysis.

The nominal Markov transition matrix $P_{0}$ used in the proposed model was set as follows:

(18) $$P_{0}=\left[\begin{array}{ccc} 0.995 & 0.005 & 0.000\\ 0.005 & 0.990 & 0.005\\ 0.000 & 0.005 & 0.995 \end{array}\right].$$

This matrix was used as a fixed nominal transition prior and was not re-optimized for individual simulation or field runs. The high diagonal values encode temporal persistence of communication states, while the low direct transition probability from the normal state to the failure state reflects the assumption that failure-level communication loss is generally preceded by a degraded communication interval. The influence of transition-matrix variations is examined through sensitivity analysis to avoid treating this nominal prior as an arbitrary fixed setting.

All simulations were conducted under identical communication observation sequences, degradation persistence settings, contextual uncertainty parameters, and RSSI attenuation conditions across all compared methods to ensure a fair comparison. The compared methods were evaluated using the same reference communication-state sequences and simulated communication observations.

The simulated communication state evolves according to the predefined probabilistic state model, while degradation events are generated stochastically to emulate transient and persistent wireless link-quality variations. Communication-level observations are represented using PRR- and RSSI-derived degradation indicators, whereas trajectory-derived contextual observations are modeled as uncertain auxiliary signals. Contextual uncertainty is represented by false-negative and false-positive probabilities, reflecting the fact that communication-sensitive regions may not always be perfectly identified during robot operation.

The main simulation settings used for communication-state evaluation are summarized in Table 2, and the default parameters of the proposed inference model are summarized in Table 3. Unless otherwise specified, the same parameter values were applied across all simulation scenarios to ensure consistent comparison.

All simulation results were averaged over 30 Monte Carlo trials to reduce stochastic variability. The simulation evaluates three major aspects of the proposed method: (i) misclassification behavior under combined signal attenuation and degradation persistence, (ii) communication-state accuracy under decreasing mean RSSI, and (iii) robustness against contextual uncertainty.

In addition to these analyses, sensitivity studies were conducted to examine the influence of degradation-evidence thresholds and transition-related parameters. Specifically, the effects of $\theta_{\mathrm{PRR}}$, $\theta_{\mathrm{RSSI}}$, the nominal transition matrix $P_{0}$, and the bounded transition coefficients were evaluated to clarify that the proposed method is not dependent on a single arbitrary parameter setting.

### 5.2. Misclassification Behavior Under Signal Attenuation and Degradation Persistence

**Figure 2:**
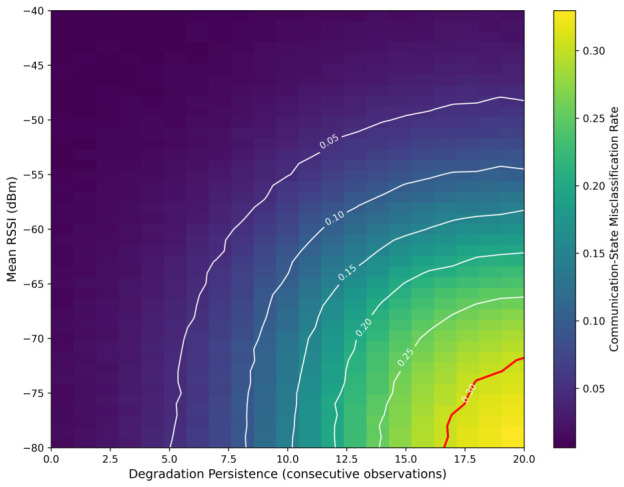
Heatmap and contour visualization of the misclassification landscape of the proposed communication-state inference framework under varying signal attenuation and degradation persistence. The horizontal axis represents degradation persistence, defined as the number of consecutive degraded communication observations, and the vertical axis represents the mean RSSI level. Color intensity indicates the communication-state misclassification rate, while contour lines show equal-error regions. The red contour highlights the 0.30 misclassification-rate level as a visual reference for high-error regions, indicating that the proposed degraded-state buffering mechanism limits excessive error growth even under sustained communication degradation.

For the misclassification landscape analysis, the mean RSSI level was varied from approximately −40 dBm to −80 dBm, while degradation persistence was varied from 0 to 20 consecutive degraded communication observations. For each grid point, the proposed inference model was evaluated under repeated stochastic degradation trials, and the resulting overall communication-state misclassification rate was averaged. This setting was designed to jointly represent communication-level attenuation and the temporal persistence of degraded observations that may occur when a remotely supervised agricultural robot traverses communication-sensitive regions. Figure 2 presents the resulting misclassification landscape of the proposed method under varying mean RSSI levels and degradation persistence.

The heatmap shows that the communication-state misclassification rate increases as signal attenuation becomes more severe and degraded observations persist for longer periods. This behavior reflects the inherent difficulty of distinguishing transient degradation from failure-level communication loss when both RSSI reduction and temporal persistence are present. Nevertheless, the contour pattern indicates that error growth remains bounded rather than increasing without limit. In particular, the high-error plateau region suggests that the proposed degraded-state buffering mechanism limits excessive failure escalation even under sustained communication degradation.

This behavior results from the probabilistic treatment of communication degradation. Instead of directly converting degraded observations into failure decisions, the proposed method first buffers uncertain evidence in the degraded state and gradually updates the failure probability only when degradation persists without sufficient contextual support. Consequently, the proposed framework reduces overconfident failure classification under transient or ambiguous communication conditions, which is essential for stable remote supervision of agricultural robots.

### 5.3. Communication-State Accuracy Under Mean RSSI Degradation

**Figure 3:**
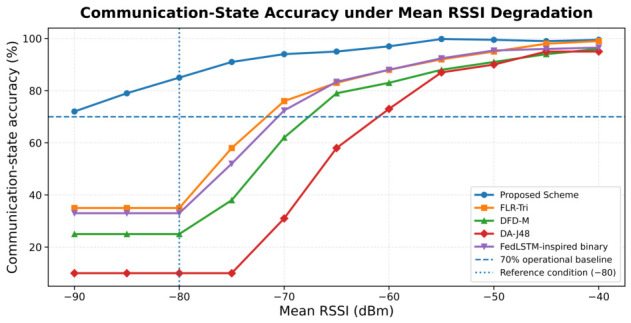
Communication-state accuracy under varying mean RSSI levels. The proposed method maintains higher accuracy as signal attenuation becomes severe, compared with the FLR-TRI, DFD-M, DA-J48, and FedLSTM-inspired binary baselines. The FedLSTM-inspired baseline represents a recent learning-based WSN sensor-fault-detection approach adapted as a binary fault-detection method. The horizontal dashed line indicates the 70% operational baseline, while the vertical dotted line marks the severe attenuation condition at −80 dBm.

RSSI is modeled as ${\mathrm{RSSI}}_{k}∼N\left(\mu ,\sigma^{2}\right)$ at each time step. Figure 3 evaluates communication-state accuracy under varying mean RSSI levels. This simulation represents signal attenuation conditions that may arise when a remotely supervised agricultural robot moves through communication-sensitive regions.

To quantitatively evaluate performance under realistic remote supervision conditions, trajectory-weighted accuracy is defined as:

(19) $$A_{\mathrm{traj}}=\frac{\sum_{k=1}^{T}\omega_{k}\cdot I\left({\hat{s}}_{k}=s_{k}\right)}{\sum_{k=1}^{T}\omega_{k}},$$

where ${\hat{s}}_{k}$ and $s_{k}$ denote the inferred and reference communication states at time step *k*, respectively, I(·) is the indicator function, and $\omega_{k}$ represents the trajectory-dependent importance weight reflecting the severity of communication degradation along the robot path. The trajectory weights were predefined from the simulated path-level degradation severity and applied identically to all compared methods.

The accuracy values reported in Figure 3 correspond to the trajectory-weighted accuracy defined in Equation (19). Trajectory-weighted accuracy is introduced to reflect realistic robot operation, where severe communication degradation may occur intermittently along the robot trajectory rather than uniformly over time. For example, a misclassification that occurs when the robot passes through a structure-dense or severe-attenuation segment is assigned a larger weight than a misclassification in an open aisle, because errors in such regions are more relevant to supervision safety and failure-prevention decisions. Because RSSI degradation varies with robot position and surrounding propagation conditions, this metric captures trajectory-dependent performance variations under remote supervision.

As shown in Figure 3, all methods exhibit reduced communication-state accuracy as the mean RSSI decreases. The proposed method maintains accuracy above the operational baseline even near the severe attenuation condition of −80 dBm, whereas the baseline methods degrade more rapidly and fall below the baseline under stronger attenuation. The FedLSTM-inspired binary baseline was added to represent a recent learning-based WSN sensor-fault-detection approach [15]. Although this baseline shows competitive behavior under mild attenuation, its accuracy decreases rapidly under severe attenuation because it does not explicitly model the intermediate degraded communication state. At −80 dBm, the proposed method maintains an accuracy of approximately 85.0%, whereas the FedLSTM-inspired binary baseline decreases to approximately 33.0%. This result indicates that degraded-state buffering prevents transient RSSI degradation from being prematurely interpreted or collapsed into binary fault-detection decisions.

The horizontal dashed line represents the 70% operational baseline, which is used as a practical lower bound for maintaining acceptable communication-state monitoring performance in the considered remote supervision scenario. The vertical dotted line at −80 dBm denotes a severe signal attenuation condition under which communication quality becomes highly unstable and conventional link-quality-based inference becomes less reliable.

The observed trends indicate that the proposed probabilistic degradation model provides more robust communication-state monitoring performance under increasing RSSI attenuation than both representative lightweight fault-detection baselines and the recent FedLSTM-inspired binary baseline.

### 5.4. Communication-State Accuracy Under Degradation Persistence

**Figure 4:**
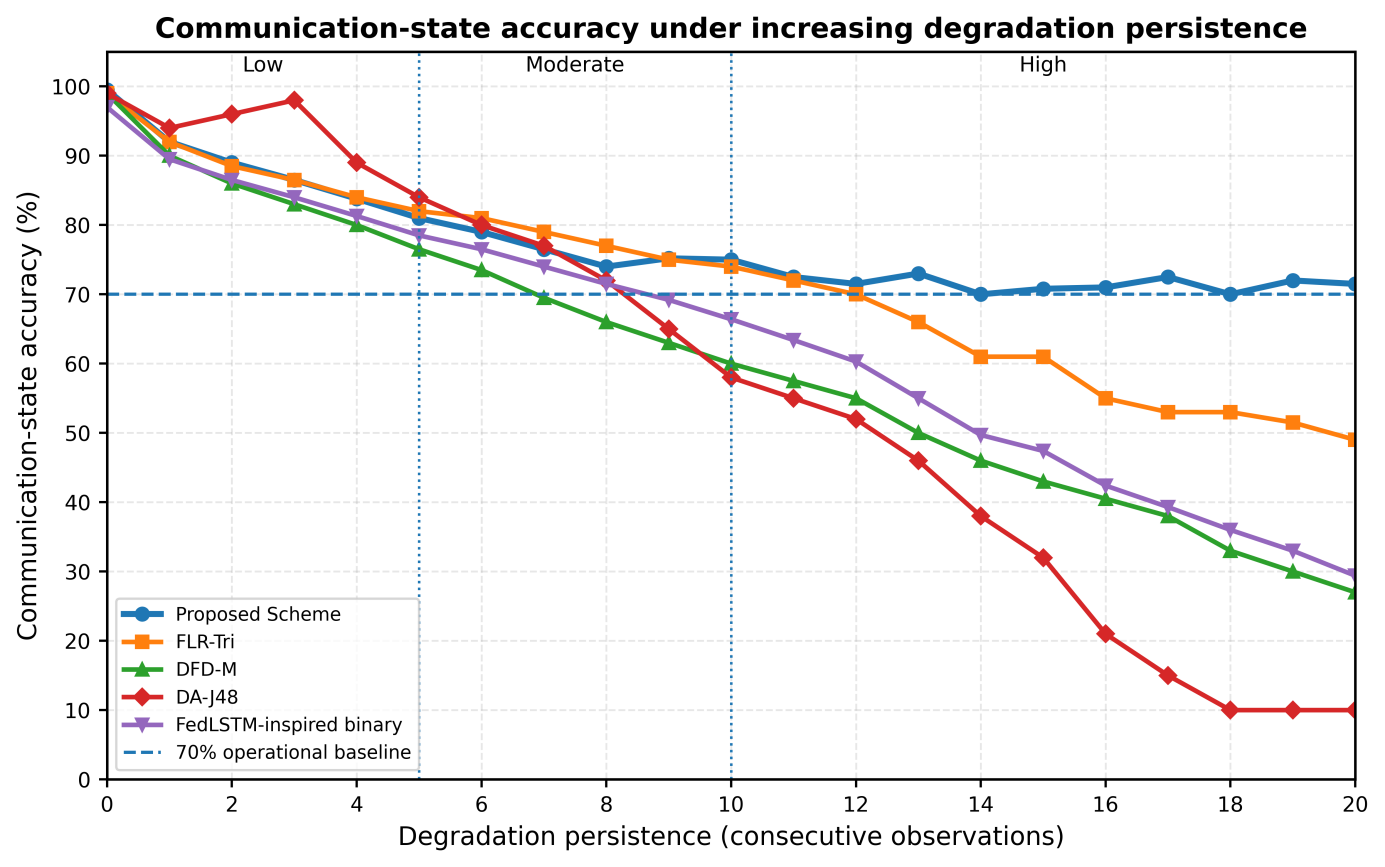
Communication-state accuracy under increasing degradation persistence. The horizontal axis represents degradation persistence, defined as the number of consecutive degraded communication observations, and the vertical axis represents communication-state accuracy. Compared with the FLR-TRI, DFD-M, DA-J48, and FedLSTM-inspired binary baselines, the proposed method exhibits slower performance degradation as communication degradation persists. The FedLSTM-inspired baseline is included as a recent learning-based WSN sensor-fault-detection baseline adapted to binary fault detection. The horizontal dashed line denotes the 70% operational baseline, and the vertical reference lines indicate the boundaries between low-, moderate-, and high-persistence regions.

While Figure 3 examines communication-state accuracy under varying mean RSSI levels, Figure 4 evaluates the effect of degradation persistence on communication-state inference performance. In this simulation, degradation persistence is defined as the number of consecutive degraded communication observations encountered during robot operation. This setting represents a remotely supervised agricultural robot that experiences sustained but potentially recoverable wireless degradation while traversing communication-sensitive regions.

As shown in Figure 4, the proposed method was compared with representative WSN fault-detection baselines: DA-J48, FLR-TRI, DFD-M, and a FedLSTM-inspired binary baseline. The DFD-M and FLR-TRI baselines are adapted from the comparative framework reported by Yang et al. [21], whereas DA-J48 follows a decision-tree-based fault-management approach for event-driven wireless sensor networks originally discussed by Ruiz et al. [22]. The FedLSTM-inspired baseline represents a recent learning-based WSN sensor-fault-detection framework [15]. These baselines are not regarded as direct models of agricultural robot communication links; rather, they are used as representative fault-detection baselines to examine how conventional and recent binary decision logic responds to communication-quality degradation. In contrast, the proposed method preserves intermediate uncertainty through probabilistic degraded-state buffering.

The horizontal reference line at 70% indicates a practical operational baseline for maintaining acceptable communication-state monitoring performance in the considered remote supervision scenario. The vertical reference lines divide the persistence range into low-, moderate-, and high-persistence regions, indicating the increasing duration of consecutive degraded communication observations.

As degradation persistence increases, the baseline methods show substantially larger performance degradation. This behavior indicates that repeated degraded observations are more likely to be collapsed into binary fault-detection decisions by methods that do not explicitly maintain a recoverable degraded communication state. The FedLSTM-inspired binary baseline also falls below the operational baseline as degradation persistence increases, reaching approximately 29.4% accuracy at the highest persistence condition. In contrast, the proposed method exhibits a slower performance decline and remains close to or above the operational baseline even in the high-persistence region, achieving approximately 71.5% accuracy at the highest persistence condition. This result suggests that the degraded-state buffering mechanism helps suppress premature failure escalation by preserving uncertain communication evidence in the degraded state before increasing the failure probability. Mechanistically, repeated degraded observations increase the degraded-state belief first, whereas failure probability grows only through the bounded degraded-to-failure transition; this prevents persistent but still recoverable attenuation from being immediately collapsed into a binary failure decision.

### 5.5. Robustness Against Contextual Uncertainty

**Figure 5:**
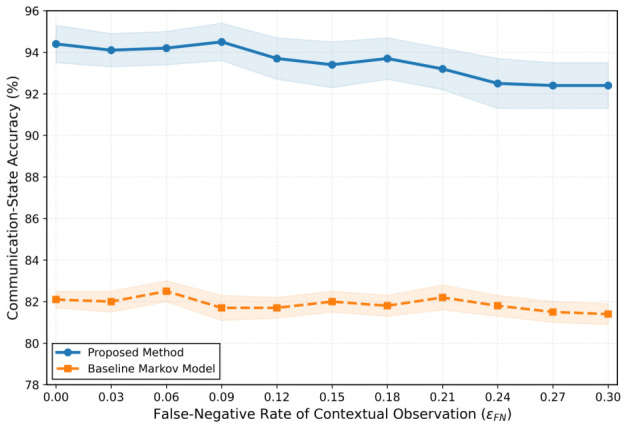
Robustness of communication-state inference under increasing contextual uncertainty, represented by the false-negative rate $\epsilon_{\mathrm{FN}}$ of trajectory-derived contextual observation. The proposed method maintains higher communication-state accuracy than the baseline Markov model as contextual observations become less reliable, indicating that bounded context-modulated transitions mitigate overconfident failure escalation under uncertain contextual information. The shaded regions indicate variability across repeated simulation trials.

Figure 5 evaluates the robustness of communication-state inference under increasing contextual uncertainty by varying the false-negative rate $\epsilon_{\mathrm{FN}}$ from 0.00 to 0.30. In this study, $\epsilon_{\mathrm{FN}}$ represents the probability that a communication-sensitive region is not correctly reflected in the trajectory-derived contextual observation.

As $\epsilon_{\mathrm{FN}}$ increases, contextual observations become less reliable, reducing their ability to support the interpretation of transient communication degradation. The baseline Markov model remains at a lower communication-state accuracy level because it does not explicitly incorporate contextual uncertainty into the transition modulation process.

In contrast, the proposed method maintains higher communication-state accuracy with only a gradual decrease as $\epsilon_{\mathrm{FN}}$ increases. This behavior indicates that the proposed framework does not use contextual information as a deterministic communication-state label. Instead, contextual information is incorporated as a bounded uncertainty-modulating signal through the contextual reliability weight $w_{k}=c_{k}\left(1-\epsilon_{\mathrm{FN}}\right)$.

This result demonstrates that the proposed bounded context-modulated transition mechanism can tolerate imperfect contextual observations while reducing overconfident failure escalation under transient or ambiguous communication degradation. In this study, $\epsilon_{\mathrm{FN}}$ is treated as the primary contextual uncertainty parameter because missed contextual observations can reduce the reliability weight $w_{k}$ and weaken the buffering effect of the degraded state. The false-positive rate $\epsilon_{\mathrm{FP}}$ is omitted from this sensitivity plot for clarity, as its effect is comparatively less direct in the proposed transition modulation mechanism.

### 5.6. Effect of Degradation-Evidence Thresholds

Because the degradation evidence $z_{k}$ is constructed from the PRR and RSSI thresholds in Equation (5), the influence of these threshold values was examined through a sensitivity analysis. The purpose of this analysis is not to identify a universally optimal threshold pair, but to verify whether the proposed probabilistic inference framework remains stable under moderate variations of the degradation-evidence criteria.

Three threshold settings were considered. The nominal setting corresponds to the default parameters used in the main simulation and field evaluation. The conservative setting uses a lower PRR threshold and a less severe RSSI threshold, resulting in fewer degradation observations. The sensitive setting uses a higher PRR threshold and a more severe RSSI threshold, resulting in more frequent degradation observations. All other model parameters were fixed to the nominal values in Table 3.

As summarized in Table 4, the proposed method maintained stable performance under moderate variations of the degradation-evidence thresholds. The nominal setting achieved an accuracy of 0.963±0.003 and a macro F1-score of 0.961±0.003. The premature failure rate remained 0.0±0.0% across the tested threshold settings, indicating that instantaneous threshold crossings were not directly converted into failure-level decisions. This result supports the role of the degraded-state buffer and bounded transition operator in suppressing premature failure escalation.

The nominal setting was selected as a balanced operating point between these two tendencies. Importantly, the proposed method does not use $\theta_{\mathrm{PRR}}$ and $\theta_{\mathrm{RSSI}}$ as direct state-decision boundaries. They are used only to generate the instantaneous degradation evidence $z_{k}$, while the final communication-state estimate is obtained through the probabilistic belief update. Therefore, moderate variations in the threshold values affect the amount of degradation evidence supplied to the model, but do not directly determine the final state sequence.

This analysis supports the interpretation that the thresholds serve as empirical evidence-generation parameters rather than deterministic communication-state classifiers. The robustness of the proposed method is mainly attributed to the degraded-state buffering and bounded transition-operator update, which prevent instantaneous threshold crossings from being immediately interpreted as failure-level communication loss.

### 5.7. Sensitivity Analysis of the Transition Operator

A second sensitivity analysis was conducted to examine the influence of transition-related parameters. This analysis directly addresses the dependence of the proposed method on the nominal Markov transition matrix $P_{0}$ and the bounded transition coefficients $\alpha_{D}\left(k\right)$ and $\alpha_{F}\left(k\right)$.

The nominal transition matrix in Equation (9) encodes temporal persistence of communication states. Because the field communication observations were sampled at 1 Hz, the diagonal elements of $P_{0}$ were set close to one to prevent evidence-free state drift between consecutive observations. This setting reflects the assumption that the latent communication state should not change abruptly unless degradation evidence is repeatedly observed. However, because the exact transition probabilities may vary depending on greenhouse layout, robot speed, gateway placement, wireless hardware, and sampling interval, it is important to examine whether the proposed method remains stable under alternative transition settings.

Three transition settings were considered. The low-persistence setting reduces the diagonal dominance of $P_{0}$, allowing more frequent state changes. The nominal setting uses the default transition matrix adopted in the main evaluation. The high-persistence setting increases the diagonal dominance of $P_{0}$, making state changes more conservative. For each setting, the same communication observation sequences and contextual inputs were used.

Table 5 shows that the proposed method maintained the same qualitative behavior under different persistence assumptions. The low-persistence setting produced the highest macro F1-score because it allowed faster adaptation to state changes in the controlled sensitivity scenarios. The high-persistence setting produced smoother but slightly delayed state evolution, resulting in a lower macro F1-score. The nominal setting provided a balanced operating point between responsiveness and temporal stability. Importantly, the premature failure rate remained 0.0±0.0% across all transition-matrix settings. This result indicates that the proposed bounded transition structure suppresses premature failure escalation even when the nominal state-persistence prior is varied.

In addition to the nominal transition matrix, the bounds of the context-modulated transition coefficients were also varied. The parameters $\alpha_{D}^{\max}$ and $\alpha_{F}^{\max}$ determine the maximum one-step transition strength toward the degraded and failure states, respectively. A larger $\alpha_{D}^{\max}$ allows faster accumulation of degradation evidence in the degraded state, whereas a larger $\alpha_{F}^{\max}$ allows faster escalation to the failure state. Therefore, these bounds affect the balance between rapid state adaptation and conservative failure escalation.

Table 6 summarizes the influence of the bounded transition coefficients. The conservative escalation setting slightly reduced accuracy and macro F1-score because the transition toward degraded and failure states was more restricted. The nominal and aggressive escalation settings produced similar performance in the controlled sensitivity scenarios, indicating that the proposed method was not overly sensitive to moderate increases in the transition bounds. Across all settings, the premature failure rate remained 0.0±0.0%. This confirms that failure escalation was effectively controlled by the degraded-state buffering mechanism and contextual modulation.

The sensitivity results in Table 5 and Table 6 indicate that the proposed method does not depend on a single arbitrary transition setting. Although the transition parameters influence the trade-off between responsiveness and temporal smoothness, the qualitative behavior of the proposed model remains consistent: degradation evidence is first accumulated in the degraded state, while failure escalation remains bounded and context-modulated. This supports the practical use of the proposed framework as an interpretable communication-state inference mechanism for remotely supervised agricultural robots.

## 6. Field Experiment and Real-World Validation

### 6.1. Experimental Environment

The simulation in Section 5 isolates RSSI attenuation, degradation persistence, and contextual uncertainty under controlled conditions. The field experiment verifies whether the same mechanism-level behavior, namely degraded-state buffering and delayed failure escalation, appears in real greenhouse link-quality measurements.

A mechanism-level field experiment was conducted in a smart greenhouse using a mobile agricultural robot and a fixed gateway. The experiment did not evaluate physical sensor hardware failure. Its purpose was to validate the proposed communication-state inference mechanism under naturally varying wireless link conditions caused by greenhouse structures, crop surroundings, narrow aisles, and robot motion.

**Figure 6:**
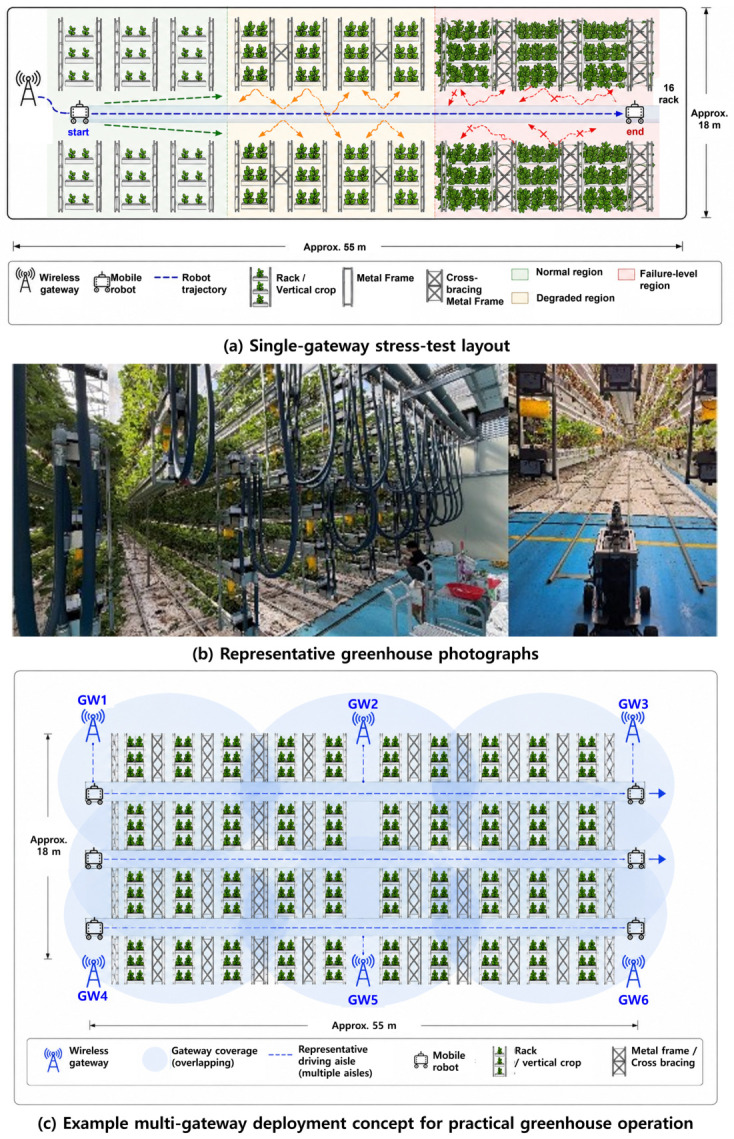
Experimental setup and multi-gateway deployment concept in the smart greenhouse. (**a**) Experimental single-gateway stress-test layout used to expose communication degradation along a representative rack-separated driving aisle; the annotated regions indicate qualitative greenhouse propagation conditions rather than a linear open-space attenuation scale. The orange wavy marks and red cross marks indicate representative degraded and failure-level communication events along the robot trajectory, respectively. (**b**) Representative greenhouse photographs of the test environment and mobile robot. (**c**) Example multi-gateway deployment concept providing overlapping coverage across rack-separated driving aisles for practical operation.

Figure 6a shows a schematic representation of the greenhouse test section and a representative robot driving aisle between vertical crop racks. The experimental section corresponded to an approximately 300-pyeong facility, equivalent to about 1000 m^2^, and was represented as an approximately 55 m × 18 m test area. The illustrated path represents one aisle among multiple rack-separated driving corridors, rather than an open-space straight-line attenuation path. The schematic includes the fixed gateway, robot start and end positions, rack arrangement, crop-canopy distribution, and metallic support structures that affect wireless propagation along the robot trajectory. During each run, the robot traveled approximately 55 m for 200 s at a low and nearly constant speed. The fixed gateway was installed near the entrance-side aisle and gateway-proximal starting region at a height comparable to the robot communication module. This single-gateway configuration was intentionally used as an experimental stress-test layout to expose communication degradation, not as a recommended deployment layout for practical greenhouse robot operation.

The normal, degraded, and failure-level regions in Figure 6a denote qualitative propagation-condition regimes rather than distance-based signal-attenuation zones. The normal region corresponds to a gateway-proximal and line-of-sight-dominant condition with relatively few obstructions. The degraded region represents partial line-of-sight blockage and increased multipath effects caused by denser crop canopy and metallic support structures. The failure-level region represents a more obstruction-dense and NLOS-prone condition, where accumulated metallic frames, cross-bracing structures, and dense vegetation increase the likelihood of severe attenuation, multipath propagation, and communication degradation. These regions should not be interpreted as exact geometric ground-truth boundaries; quantitative evaluation labels were assigned according to the reference-regime definition in Section 6.4.

Figure 6b shows the actual greenhouse aisle, vertical rack structures, metallic frames, and cultivation infrastructure that produced attenuation, multipath propagation, and intermittent obstruction during robot-to-gateway communication. Figure 6c presents an example multi-gateway deployment concept for practical greenhouse robot operation. In this concept, multiple gateways provide overlapping coverage across rack-separated driving aisles, reducing communication blind spots caused by metallic structures, dense crop canopies, and NLOS-prone propagation. The layout is a practical deployment guideline derived from the observed degradation pattern, not an optimized gateway-placement result. Gateway-placement optimization is beyond the scope of this study, but the proposed inference framework can support deployment planning by identifying regions where additional communication coverage may be required.

### 6.2. System Configuration

The experimental system consisted of a mobile agricultural robot equipped with wireless communication and link-quality logging modules, and a fixed gateway node deployed inside the greenhouse. During operation, the robot periodically exchanged packets with the fixed gateway while traversing the predefined greenhouse path. The gateway served as the reference receiver for collecting packet-level communication measurements, including packet reception ratio (PRR) and received signal strength indicator (RSSI).

In this experiment, the robot-to-gateway link was used as a representative communication link between a remotely supervised agricultural robot and a supervision infrastructure. The communication module was configured to log real-time link-quality variations during robot motion, rather than to emulate artificial packet loss or synthetic channel degradation. Thus, the collected data reflect naturally occurring communication fluctuations observed during greenhouse robot operation. The experimental system configuration and data acquisition conditions are summarized in Table 7.

### 6.3. Data Acquisition Process

All communication data were directly collected from a physical greenhouse deployment during robot operation. The dataset was obtained from packet-level communication logs recorded while the robot moved through predefined greenhouse paths. At each time step, the following communication metrics were recorded:

- Packet reception ratio (PRR), computed over a sliding window of transmitted packets.
- Received signal strength indicator (RSSI), measured for received packets.

All data were sampled at 1 Hz, which is consistent with the operational constraints of embedded robotic systems and lightweight communication monitoring modules. The robot traversed predefined paths between crop rows at approximately constant speed, while communication measurements were continuously logged throughout each run. The data acquisition process captured link-quality variations caused by crop-canopy attenuation, metallic greenhouse structures, irrigation facilities, multipath propagation, and local attenuation effects induced by robot motion.

No artificial noise injection, synthetic packet-loss generation, or simulated channel degradation was applied to the field data. The collected dataset therefore reflects naturally occurring fluctuations and degradation patterns observed in a real greenhouse deployment.

To ensure temporal consistency, all measurements were timestamped and synchronized with the robot trajectory. This enabled the relationship between communication quality variations and spatial environmental conditions encountered during operation to be analyzed. Each experimental run consisted of one complete traversal of the predefined greenhouse path. A total of five repeated runs were conducted along the same path to maintain consistent environmental exposure while allowing natural variations in link quality caused by multipath propagation, plant-induced attenuation, and robot motion.

**Figure 7:**
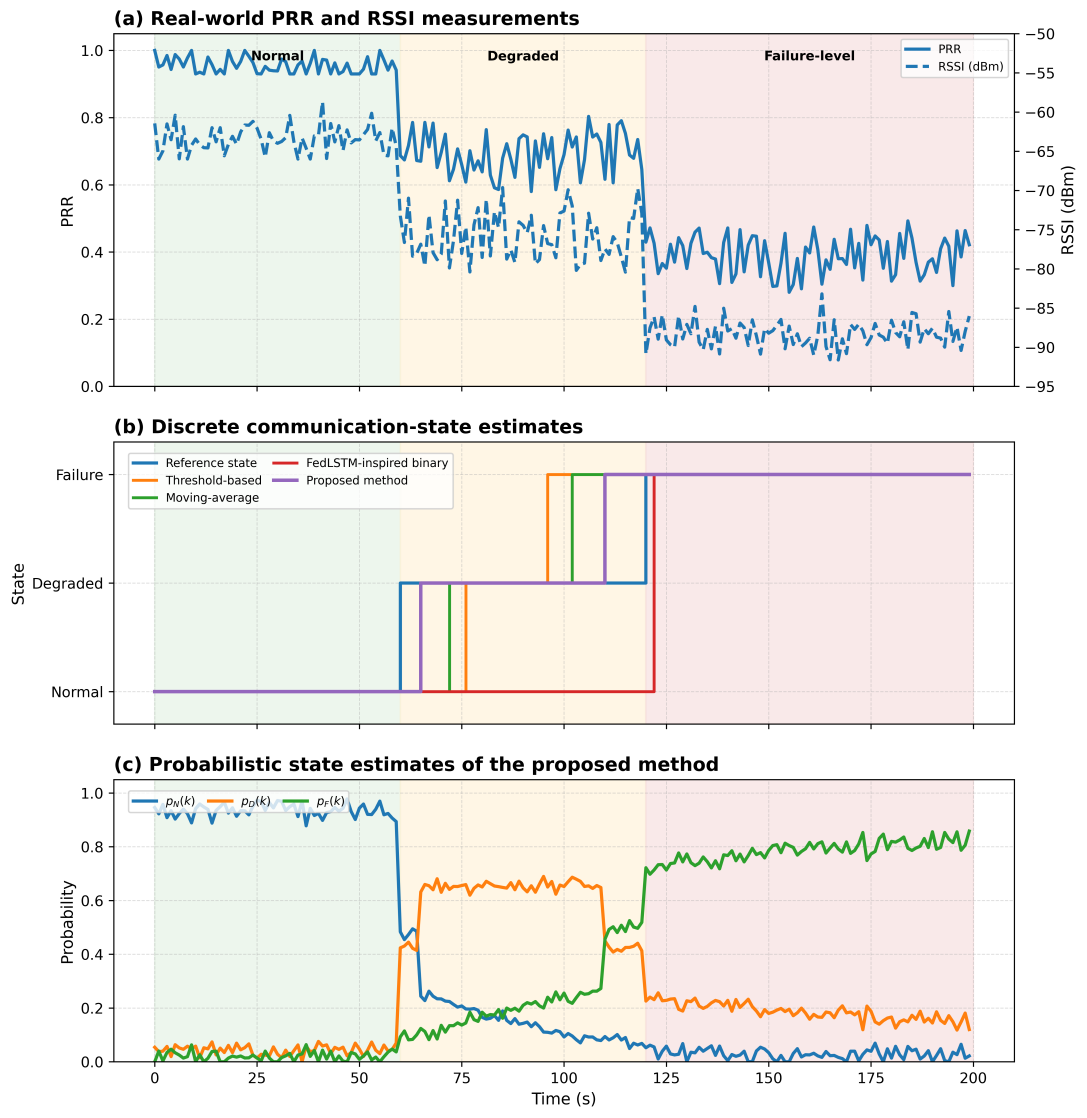
Representative field validation results obtained from one complete greenhouse run of a remotely supervised agricultural robot. (**a**) Real-world PRR and RSSI measurements collected along the robot trajectory, with shaded regions indicating reference communication regimes primarily annotated from predefined trajectory-dependent greenhouse regions. PRR and RSSI measurements were used only for post hoc consistency verification of the annotated regimes, rather than as direct threshold rules for assigning reference labels. (**b**) Discrete communication-state estimates produced by the threshold-based method, moving-average method, FedLSTM-inspired binary baseline, and proposed method, compared with the reference communication state. The FedLSTM-inspired baseline represents a recent learning-based WSN sensor-fault-detection approach adapted as a binary fault-detection baseline; therefore, it does not explicitly output the intermediate degraded communication state. (**c**) Probabilistic state estimates of the proposed method, showing the evolution of normal, degraded, and failure-state probabilities over time. The increase in degraded-state probability during the transition interval indicates that the proposed method buffers uncertain communication degradation before escalating to failure-level communication loss.

The representative result shown in Figure 7 corresponds to one complete field run and is used for qualitative time-series interpretation. The quantitative metrics were computed as the mean and standard deviation across the five repeated runs and are reported in the quantitative evaluation section. This separation is important because Figure 7 illustrates the temporal behavior of the proposed inference mechanism, whereas the repeated-run performance is summarized separately in the quantitative evaluation section.

Because the objective of the field experiment was to validate the communication-state inference mechanism rather than the standalone performance of a vision or perception model, trajectory-derived environmental context was used as a surrogate contextual observation. This setting preserves the intended role of context in the proposed framework, namely confidence modulation rather than direct communication-state determination.

### 6.4. Reference Communication-State Definition

To ensure a fair evaluation, the reference communication-state labels were defined independently from the direct thresholding rules used by the baseline methods. The labeling process was primarily based on the robot trajectory and known environmental regions within the greenhouse. The qualitative propagation-condition regimes illustrated in Figure 6a clarify the physical basis of the normal, degraded, and failure-level candidate zones, including line-of-sight availability, canopy obstruction, metallic-frame density, and multipath-prone conditions. PRR and RSSI measurements were used only for post hoc consistency verification, rather than as direct decision thresholds for label assignment.

The labeling process followed a two-stage procedure:

1. **Environment-driven annotation:** The robot trajectory was segmented into distinct communication regimes based on known physical propagation conditions in the greenhouse, including relatively open line-of-sight regions, partially obstructed rack-row regions, and structure-dense regions where accumulated racks, metallic support frames, cross-bracing structures, and dense crop canopies increased line-of-sight blockage and severe multipath propagation.
2. **Signal consistency verification:** After the initial environment-driven annotation, PRR and RSSI measurements were analyzed to verify that the annotated regions exhibited statistically consistent communication characteristics.

Importantly, the observed PRR and RSSI ranges were not used as predefined thresholds for assigning reference labels. Instead, they are reported as descriptive statistics of the annotated communication regimes. This strategy avoids defining reference labels directly from the same PRR and RSSI values used as model inputs, while still enabling evaluation of communication-state inference under real greenhouse operating conditions.

The annotated communication regimes are summarized as follows:

- **Normal communication regime:** The robot operated in gateway-proximal or relatively open aisle regions with favorable line-of-sight conditions and limited structural occlusion, as represented by the normal regime in Figure 6a. In this regime, communication was consistently reliable, with high PRR values typically within the range of 0.95–1.0 and relatively strong RSSI values around −60 dBm.
- **Degraded communication regime:** The robot traversed regions with partial line-of-sight obstruction caused by rack structures, denser crop canopies, and metallic supports, as represented by the degraded regime in Figure 6a. In this regime, communication exhibited intermittent degradation, with moderate PRR values approximately within the range of 0.6–0.8 and fluctuating RSSI values around −70 to −80 dBm.
- **Failure communication regime:** The robot operated in severely attenuated regions where sustained signal degradation occurred due to accumulated metallic frames, intersecting rack structures, cross-bracing structures, dense crop canopies, and NLoS-prone propagation geometry, as represented by the failure-level regime in Figure 6a. This regime was characterized by persistently low PRR values, approximately within the range of 0.3–0.5, and weak RSSI values around −85 to −90 dBm. In this study, this state denotes a persistent link-level communication failure condition, not irreversible physical failure of sensor hardware.

Based on the predefined environmental regions along the robot trajectory, the trajectory was annotated into normal, degraded, and failure communication regimes. The schematic regimes in Figure 6a indicate that this segmentation was grounded in physical propagation conditions rather than arbitrary distance-only partitioning. Transition overlap regions were explicitly considered to account for gradual spatial changes between adjacent propagation conditions. The reference labels therefore represent operational communication-state regimes observed during robot operation, rather than physical hardware failure states or arbitrarily imposed time segments.

The final reference labels were assigned based on trajectory-dependent environmental regions, while communication signal characteristics were used only to verify the consistency of the assigned regimes after annotation. Although the labeling process used controlled knowledge of the experimental environment, the communication measurements themselves were entirely derived from real-world observations. This labeling strategy is appropriate because the proposed framework aims to identify operational communication states rather than diagnose the physical root cause of hardware malfunction.

### 6.5. Representative Field Validation of Communication-State Inference

Figure 7 presents a representative field-validation result obtained from one complete greenhouse run. Unlike the simulation results, which were designed to analyze communication-state inference behavior under controlled uncertainty conditions, this figure illustrates how the proposed framework and the compared baselines behave on real-world communication measurements acquired during robot motion. The FedLSTM-inspired binary baseline was included in Figure 7 to maintain consistency with the expanded comparisons in Figure 3 and Figure 4 and the adapted field-baseline comparison presented in Section 6.7.

As shown in Figure 7a, both PRR and RSSI vary over time as the robot traverses different environmental regions. The observed communication quality does not change as a perfectly discrete step function; instead, intermediate transition intervals appear between the normal, degraded, and failure communication regimes. This behavior supports the need for probabilistic communication-state inference rather than direct threshold-based failure judgment.

Figure 7b compares the discrete communication-state estimates of the threshold-based method, moving-average method, FedLSTM-inspired binary baseline, and proposed method against the reference communication state. The threshold-based and moving-average methods exhibit either abrupt or delayed transitions depending on the communication fluctuation pattern. The FedLSTM-inspired binary baseline provides an additional comparison with a recent learning-based WSN fault-detection approach; however, because it is adapted as a binary fault-detection baseline, it tends to collapse intermediate communication conditions into normal-like or failure-like decisions rather than explicitly maintaining the degraded communication state. By contrast, the proposed method more consistently follows the reference communication-state evolution while preserving an intermediate degraded interpretation during uncertain intervals.

Figure 7c further shows the probabilistic state evolution of the proposed method. When communication degradation first appears, the degraded-state probability increases before the failure probability becomes dominant. This confirms that the proposed framework does not immediately interpret short-term communication degradation as failure-level communication loss. Instead, uncertain degradation is buffered in the degraded state and escalated only when the degraded pattern persists.

Figure 7 is presented as a representative qualitative example from one complete field run. The quantitative comparison across repeated runs is summarized separately in Table 9, while the expanded comparison with adapted fault-detection baselines, including the FedLSTM-inspired binary baseline, is reported in Table 10. This run was selected to illustrate the typical temporal transition behavior observed during greenhouse operation, rather than to serve as a standalone performance evaluation.

### 6.6. Quantitative Evaluation on Field Data

Unlike the simulation setup, the field experiment does not impose explicit temporal constraints such as $\tau_{\lim}$, $\tau_{\mathrm{rec}}$, or $e_{m}$. Instead, the evaluation is conducted directly on real-world communication measurements, allowing the proposed probabilistic model to operate without externally enforced persistence rules.

For quantitative evaluation, the probabilistic output of the proposed method was converted into discrete states using the maximum posterior probability criterion. This conversion was applied only for metric calculation; the proposed framework itself preserves continuous state probabilities during inference. The threshold-based baseline classified communication states using predefined PRR criteria, whereas the moving-average baseline applied temporal smoothing to PRR measurements before state classification. All methods were evaluated using the same input data and reference labels to ensure a consistent comparison.

The threshold-based and moving-average baselines were implemented as representative lightweight communication-state estimation methods based on packet-level link-quality indicators. The same parameter settings were applied across all repeated field runs to avoid run-specific tuning. For the proposed method, the parameters listed in Table 3 were fixed before evaluation and were not re-optimized for individual runs.

Table 8 summarizes the operational baselines used in the primary field evaluation, whereas Section 6.7 extends the comparison to adapted fault-detection baselines derived from related WSN studies.

State-wise accuracy was computed for the normal, degraded, and failure intervals defined in Section 6.4. In addition to overall accuracy and macro F1-score, two operational metrics were considered:

- **Detection delay:** Time difference between the reference degradation onset and the detected transition into the degraded communication state.
- **Premature failure rate:** Fraction of time steps incorrectly classified as failure-level communication loss during reference degraded intervals.

The quantitative metrics summarized in Table 9 were averaged over five repeated field runs conducted along the same predefined greenhouse path. The reported values include the mean and standard deviation to reflect run-to-run variability induced by natural greenhouse propagation dynamics and stochastic communication behavior.

To examine whether the reduction in premature failure rate was statistically meaningful, a paired comparison was conducted across the five repeated field runs. The proposed method was compared with the threshold-based and moving-average baselines using the same run-wise premature failure rates. Compared with the threshold-based method, the proposed method reduced the premature failure rate by an average of 24.33 percentage points, and the paired *t*-test indicated that this reduction was statistically significant (t(4)=21.53, $p=2.75\times 10^{-5}$). Compared with the moving-average method, the proposed method reduced the premature failure rate by an average of 13.67 percentage points, which was also statistically significant (t(4)=41.00, $p=2.11\times 10^{-6}$). These results show that the reduction in premature failure decisions was consistent across the repeated greenhouse runs rather than being caused by a single favorable trial.

The proposed method achieved the highest overall accuracy and macro F1-score among the compared methods. The largest improvement occurred in the degraded communication regime and transition overlap regions, where conventional threshold-based or smoothed-threshold methods tended to either delay detection or prematurely escalate the state to failure-level communication loss. The proposed method achieved a degraded-state accuracy of 0.717±0.024, compared with 0.273±0.042 for the threshold-based method and 0.467±0.026 for the moving-average method. This confirms that the proposed method is particularly effective in ambiguous transition periods, rather than merely improving performance in clearly normal or clearly failure-level conditions.

The proposed method also reduced detection delay and premature failure rate. The reduced delay indicates that the proposed model can identify the onset of communication degradation earlier than the baseline methods. The lower premature failure rate indicates that the degraded-state buffer prevents degraded intervals from being immediately interpreted as failure-level communication loss. These findings are consistent with the intended role of the degraded state: communication degradation in remotely supervised agricultural robot operation should be treated as probabilistic evidence with intermediate uncertainty, rather than as an immediate binary indication of failure.

These results should be interpreted as mechanism-level validation under real greenhouse link-quality variations, while broader deployment-level validation is discussed in Section 6.8.

### 6.7. Field Comparison with Adapted Fault-Detection Baselines

To provide a systematic quantitative comparison with representative methods discussed in the review section, fault-detection baselines derived from both foundational and recent WSN studies were adapted and re-evaluated using the same greenhouse field dataset, reference communication-state labels, and evaluation metrics. The adapted baselines include DA-J48, FLR-TRI, and DFD-M as representative lightweight fault-detection methods, as well as a FedLSTM-inspired baseline representing a recent learning-based WSN sensor-fault-detection framework [15]. A direct numerical comparison with the originally reported results of these studies would be inappropriate because the datasets, fault definitions, sensing modalities, and evaluation objectives differ substantially from those of the present greenhouse robot-supervision task. Therefore, the comparison was conducted as a same-dataset adapted evaluation using the field data, reference communication-state labels, and metrics adopted in this study.

Because the original DA-J48-, FLR-TRI-, DFD-M-, and FedLSTM-based methods were developed mainly for binary fault/non-fault, sensor-data fault-detection, or sensor-node fault-detection problems, the adapted baselines were implemented as binary fault-detection methods. Specifically, these baselines classified each time step as either normal communication or failure-level communication loss, without explicitly introducing a degraded communication state. This setting preserves the original operational role of the cited fault-detection methods and avoids embedding the main contribution of the proposed method, namely the intermediate degraded-state buffer, into the baselines. The parameters of the adapted baselines were fixed before field evaluation and were not optimized using the field reference labels. This condition was imposed to prevent overly optimistic post hoc threshold fitting to the greenhouse dataset. All methods were evaluated using the same five repeated field runs and the same reference labels. The detection delay metric was treated as not applicable for the binary adapted baselines because these methods do not output an explicit degraded state and therefore cannot define a transition time into the degraded communication regime.

As shown in Table 10, the adapted binary fault-detection baselines were limited under the three-state communication-state evaluation. Although these baselines can represent clearly normal or clearly failure-level communication conditions, they cannot explicitly maintain an intermediate degraded state. Consequently, their degraded-state accuracy was zero, and the detection delay into the degraded communication regime could not be computed. The repeated accuracy values observed for several adapted binary baselines arise from the class composition of the three-state reference labels and the absence of an explicit degraded-state output, not from constant-value assignment. This finding supports the proposed formulation: in remote agricultural robot supervision, transient degraded communication should not be collapsed into a binary fault/non-fault decision because such a representation cannot distinguish recoverable degradation from persistent failure-level communication loss.

The FedLSTM-inspired baseline was included to represent a recent learning-based WSN fault-detection approach. Although this binary baseline produced no premature failure decisions during reference degraded intervals, this result should not be interpreted as successful degraded-state recognition. Rather, it reflects the limitation of a binary fault-detection formulation: intermediate degraded communication conditions are collapsed into non-degraded or fault-level decisions instead of being explicitly maintained as a recoverable operational state.

Compared with the adapted fault-detection baselines, the proposed method achieved the highest overall accuracy and macro F1-score while explicitly recognizing the degraded communication regime. The proposed method achieved a degraded-state accuracy of 0.717±0.024 and a detection delay of 6.2±0.8 s, whereas the binary adapted baselines could not provide meaningful degraded-state detection. In addition, the premature failure rate of the proposed method was reduced to 18.0±1.4%, which was lower than those of the threshold-based, moving-average, DA-J48-inspired, FLR-TRI-inspired, and DFD-M-inspired baselines. Although the FedLSTM-inspired binary baseline produced a premature failure rate of 0.0±0.0%, this value reflects the absence of explicit degraded-state output rather than successful recognition of degraded communication intervals. These results show that the proposed degraded-state buffering mechanism provides an operational advantage for remote supervision by reducing premature escalation to failure-level communication loss while preserving explicit recognition of degraded but recoverable communication intervals.

### 6.8. Discussion and Limitations

The field experiment used five repeated runs along one predefined greenhouse path. The results therefore provide mechanism-level evidence rather than a complete statistical generalization across agricultural wireless environments. Within this scope, the field data show that degraded-state buffering can handle gradual and ambiguous communication-state transitions using real PRR and RSSI measurements.

The same-dataset comparison with adapted fault-detection baselines in Section 6.7 clarifies the main advantage of the proposed formulation. Binary fault-detection logic, including the FedLSTM-inspired baseline, can represent clearly normal or failure-level conditions but cannot explicitly maintain a recoverable degraded communication state. The proposed method improves aggregate metrics while preserving this intermediate state during ambiguous intervals.

The current validation is limited to a single robot-to-gateway link in one smart greenhouse. Communication behavior may change with greenhouse layout, crop density, gateway placement, antenna configuration, wireless protocol, and robot trajectory. Although Figure 6c illustrates an example multi-gateway deployment concept for practical operation, this study does not optimize gateway locations or evaluate network handover, routing, or multi-gateway coordination. Future experiments should include orchards, open-field environments, quantitatively evaluated multi-gateway deployments, and multi-robot remote supervision scenarios.

This study does not aim to optimize physical-layer throughput, routing, spectrum use, or wireless protocol design. Its contribution is a sensor-derived state-inference framework that uses link-quality observations and trajectory context to support communication-aware robot supervision. This scope aligns with sensing-based monitoring and robotic supervision rather than communication-protocol optimization.

## 7. Conclusions

This paper presented a probabilistic communication-state inference framework for remotely supervised agricultural robots under intermittent wireless link degradation. The method represents the robot-to-gateway link using normal, degraded, and failure states. The degraded state serves as an uncertainty buffer that prevents short-term packet loss or RSSI attenuation from being treated as immediate failure-level communication loss.

The framework combines PRR, RSSI, and trajectory-derived context through a bounded context-modulated transition mechanism. Communication observations provide primary evidence, while context adjusts transition confidence without directly determining the state. This structure preserves uncertainty during transient degradation and increases failure probability only when degradation persists.

Simulation results showed robust communication-state accuracy under signal attenuation, degradation persistence, and contextual uncertainty. Field validation with real greenhouse communication data achieved an accuracy of 0.915±0.007 and a macro F1-score of 0.907±0.008, while reducing the premature failure rate to 18.0±1.4%. Paired tests over five repeated runs confirmed statistically significant reductions in premature failure rate compared with the threshold-based baseline (24.33 percentage points, $p=2.75\times 10^{-5}$) and the moving-average baseline (13.67 percentage points, $p=2.11\times 10^{-6}$). Same-dataset comparisons with adapted fault-detection baselines, including a FedLSTM-inspired baseline, showed that binary fault-detection logic cannot explicitly represent recoverable degraded communication intervals.

The proposed method is lightweight and suitable for real-time implementation on embedded agricultural robot platforms. Future work will extend validation to diverse greenhouse layouts, orchards, open fields, wireless protocols, gateway configurations, and multi-robot supervision scenarios. Mission-level metrics such as operation continuity, safe-stop reduction, communication recovery time, and operator intervention frequency will also be considered.

These findings support probabilistic degradation modeling as a sensor-derived state-inference approach for communication-aware remote supervision of agricultural robots.

## Figures and Tables

**Table 1:** List of key mathematical symbols and variables.

Symbol	Description
$S_{N},S_{D},S_{F}$	Normal, degraded, and failure-level communication states
$S_{k}$	Latent communication state at time step *k*
${\hat{S}}_{k}$	Estimated communication state at time step *k*
$p_{k}$	Communication-state belief vector at time step *k*
$q_{k}$	Predicted belief after nominal Markov prediction
$P_{0}$	Nominal row-stochastic Markov transition matrix
$R_{k}$	Bounded context-modulated transition operator
$z_{k}$	Communication degradation evidence derived from PRR and RSSI
$c_{k}$	Trajectory-derived contextual indicator
$C_{k}$	Latent communication-sensitive context condition
$w_{k}$	Contextual reliability weight, $w_{k}=c_{k}\left(1-\epsilon_{\mathrm{FN}}\right)$
$\theta_{\mathrm{PRR}}$	PRR threshold for constructing degradation evidence
$\theta_{\mathrm{RSSI}}$	RSSI threshold for constructing degradation evidence
$\epsilon_{\mathrm{FN}}$	False-negative rate of contextual observation
$\epsilon_{\mathrm{FP}}$	False-positive rate of contextual observation
$\gamma_{D}$	Normal-to-degraded transition modulation coefficient
$\gamma_{F}$	Degraded-to-failure transition modulation coefficient
λ	Context modulation scaling factor
$\alpha_{D}\left(k\right)$	Bounded normal-to-degraded transition coefficient
$\alpha_{F}\left(k\right)$	Bounded degraded-to-failure transition coefficient
$\alpha_{D}^{\max}$	Upper bound of $\alpha_{D}\left(k\right)$
$\alpha_{F}^{\max}$	Upper bound of $\alpha_{F}\left(k\right)$
clip(·)	Saturation operator limiting a value to a predefined interval

**Table 2:** Simulation settings for communication-state evaluation.

Parameter	Value	Description
Sampling interval	1 s	Communication observation period for discrete-time state updates
Mean RSSI range for misclassification landscape analysis	−40 to −80 dBm	Signal attenuation range used in the misclassification landscape analysis
Mean RSSI range for RSSI-based accuracy comparison	−40 to −90 dBm	Signal attenuation range used in the RSSI-based accuracy comparison
RSSI step size	1 dB for misclassification analysis; 5 dB for RSSI sweep analysis	Grid resolution for RSSI sweep experiments
Degradation persistence (*d*)	0–20 observations	Number of consecutive degraded communication observations
Contextual uncertainty ($\epsilon_{\mathrm{FN}}$)	0.00–0.30	False-negative rate range of contextual observation used in contextual uncertainty analysis
Contextual uncertainty step	0.03	Step size for $\epsilon_{\mathrm{FN}}$ sensitivity analysis
Monte Carlo trials	30 trials	Number of stochastic trials used for each grid point in the misclassification landscape
Operational baseline	70%	Practical lower-bound reference for acceptable communication-state accuracy
Severe attenuation condition	−80 dBm	Reference RSSI level indicating severe wireless signal attenuation
Initial belief state	[1,0,0]	Initial probability assigned to normal, degraded, and failure states

**Table 3:** Default parameters of the proposed probabilistic inference model.

Symbol	Definition	Nominal Value	Selection Rationale
$p_{0}$	Initial belief vector	[1,0,0]	Initial operation starts from a normal communication region
$P_{0}$	Nominal transition matrix	Equation (9)	1 Hz temporal persistence prior; sensitivity-tested
$\theta_{\mathrm{PRR}}$	PRR threshold for constructing $z_{k}$	0.70	Empirical boundary for degradation evidence
$\theta_{\mathrm{RSSI}}$	RSSI threshold for constructing $z_{k}$	−80 dBm	Empirical attenuation boundary in the greenhouse testbed
$\gamma_{D}$	Normal-to-degraded modulation coefficient	0.10	Nominal coefficient for degraded-state buffering
$\gamma_{F}$	Degraded-to-failure modulation coefficient	0.07	Conservative coefficient for failure escalation
λ	Context modulation scaling factor	0.50	Moderate contextual influence on transition modulation
$\epsilon_{\mathrm{FN}}$	False-negative rate of contextual observation	0.10	Default contextual uncertainty setting
$\epsilon_{\mathrm{FP}}$	False-positive rate of contextual observation	0.05	General contextual observation-error parameter
$\alpha_{D}^{\max}$	Upper bound of $\alpha_{D}\left(k\right)$	0.20	Prevents excessive one-step transition to the degraded state
$\alpha_{F}^{\max}$	Upper bound of $\alpha_{F}\left(k\right)$	0.10	Suppresses abrupt one-step escalation to the failure state
${\hat{S}}_{k}$	Discrete state decision rule	$\arg\max_{i}p_{i}\left(k\right)$	Used only for metric calculation and supervisory decision support

**Table 4:** Sensitivity analysis results under different PRR and RSSI degradation-evidence threshold settings.

Setting	$\theta_{\mathrm{PRR}}$	$\theta_{\mathrm{RSSI}}$	Accuracy	Macro F1-Score	Premature Failure Rate (%)
Conservative	0.65	−75 dBm	0.964±0.002	0.962±0.002	0.0±0.0
Nominal	0.70	−80 dBm	0.963±0.003	0.961±0.003	0.0±0.0
Sensitive	0.75	−85 dBm	0.977±0.003	0.976±0.003	0.0±0.0

**Table 5:** Sensitivity analysis of the nominal transition matrix.

Setting	$p_{\mathrm{NN}}$	$p_{\mathrm{DD}}$	$p_{\mathrm{FF}}$	Macro F1-Score	Premature Failure Rate (%)
Low persistence	0.990	0.980	0.990	0.972±0.002	0.0±0.0
Nominal	0.995	0.990	0.995	0.961±0.003	0.0±0.0
High persistence	0.998	0.996	0.998	0.947±0.002	0.0±0.0

**Table 6:** Sensitivity analysis of bounded transition coefficients.

Setting	$\alpha_{D}^{\max}$	$\alpha_{F}^{\max}$	Accuracy	Macro F1-Score	Premature Failure Rate (%)
Conservative escalation	0.15	0.05	0.954±0.002	0.953±0.002	0.0±0.0
Nominal	0.20	0.10	0.963±0.003	0.961±0.003	0.0±0.0
Aggressive escalation	0.25	0.15	0.963±0.003	0.961±0.003	0.0±0.0

**Table 7:** Experimental system configuration and data acquisition conditions.

Item	Description
Mobile platform	AgileX Scout Mini-based mobile agricultural robot platform
Operating environment	Smart greenhouse with crop rows, metallic frames, and narrow corridors
Communication setup	Mobile robot-to-fixed gateway wireless communication link
Logged communication metrics	PRR and RSSI
Gateway node	Fixed reference receiver deployed inside the greenhouse
Sampling rate	1 Hz
Experiment duration	200 s per run
Robot motion	Representative aisle navigation between vertical crop racks at approximately constant speed
Reference state labels	Normal, degraded, and failure communication states
Data type	Real-world link-quality measurements without synthetic noise injection
Evaluation target	Communication-state inference, not physical hardware failure diagnosis
Number of repeated runs	Five repeated runs along the same predefined greenhouse path

**Table 8:** Implementation conditions of the compared communication-state estimation methods.

Method	Input Variables	Decision Rule	Parameter Policy
Threshold-based	PRR	Direct PRR-based state thresholding	Fixed across all runs
Moving average	PRR	Smoothed PRR-based state thresholding	Fixed window size across all runs
Proposed method	PRR, RSSI, context	Probabilistic belief update	Fixed parameters in Table 3

**Table 9:** Quantitative performance comparison on greenhouse communication data averaged over five repeated runs.

Method	Accuracy	Macro F1-Score	Degraded-State Acc.	Detection Delay (s)	Premature Failure Rate (%)
Threshold-based	0.782±0.013	0.720±0.022	0.273±0.042	18.2±1.5	42.3±1.9
Moving average	0.840±0.008	0.811±0.011	0.467±0.026	13.0±0.7	31.7±1.7
Proposed method	0.915±0.007	0.907±0.008	0.717±0.024	6.2±0.8	18.0±1.4

**Table 10:** Same-dataset field comparison with adapted fault-detection baselines derived from foundational and recent related studies. The adapted DA-J48-, FLR-TRI-, DFD-M-, and FedLSTM-inspired baselines were implemented as binary fault-detection methods because the original approaches do not explicitly model an intermediate degraded communication state for agricultural robot supervision.

Method	Accuracy	Macro F1-Score	Degraded-State Acc.	Detection Delay (s)	Premature Failure Rate (%)
Threshold-based	0.782±0.013	0.720±0.022	0.273±0.042	18.2±1.5	42.3±1.9
Moving average	0.840±0.008	0.811±0.011	0.467±0.026	13.0±0.7	31.7±1.7
DA-J48-inspired binary baseline	0.700±0.000	0.548±0.001	0.000±0.000	N/A	53.0±5.9
FLR-TRI-inspired binary baseline	0.700±0.000	0.557±0.002	0.000±0.000	N/A	76.0±4.2
DFD-M-inspired binary baseline	0.700±0.000	0.548±0.001	0.000±0.000	N/A	24.3±7.9
FedLSTM-inspired binary baseline	0.689±0.002	0.548±0.001	0.000±0.000	N/A	0.0±0.0
Proposed method	0.915±0.007	0.907±0.008	0.717±0.024	6.2±0.8	18.0±1.4

## Data Availability

The data presented in this study are available on request from the corresponding author. The data are not publicly available due to institutional and project-related restrictions.
